# Medical, Genomic, and Evolutionary Aspects of the Peptide Sharing between Pathogens, Primates, and Humans

**DOI:** 10.1055/s-0040-1716334

**Published:** 2020-08-31

**Authors:** Darja Kanduc, Yehuda Shoenfeld

**Affiliations:** 1 Department of Biosciences, Biotechnologies, and Biopharmaceutics, University of Bari, Bari, Italy; 2 Zabludowicz Center for Autoimmune Diseases, Sheba Medical Center, University School of Medicine, Tel-Aviv University, Tel-Hashomer, Israel; 3 I.M. Sechenov First Moscow State Medical University of the Ministry of Health of the Russian Federation, Sechenov University, Moscow, Russia

**Keywords:** peptide sharing, cross-reactivity, autoimmunity, nonhuman primates, preclinical tests

## Abstract

Comparing mammalian proteomes for molecular mimicry with infectious pathogens highlights the highest levels of heptapeptide sharing between pathogens and human, murine, and rat proteomes, while the peptide sharing level is minimal (or absent) with proteomes from nonhuman primates such as gorilla, chimpanzee, and rhesus macaque. From the medical point of view, the data might be useful to clinicians and vaccinologists to develop and evaluate immunomodulatory and immunotherapeutic approaches. As a matter of fact, primates seem to be unreliable animal models for revealing potential autoimmune events in preclinical testing of immunotherapies. In terms of genomics, the scarce or absent peptide sharing between pathogens and primates versus the massive peptide sharing existing between pathogens and humans lets foresee mechanisms of pathogen sequence insertion/deletion/alteration that have differently operated in mammals over evolutionary timescales. Why and how the human genome has been colonized by pathogen sequences and why and how primates escaped such a colonization appears to be the new scientific challenge in our efforts to understand not only the origin of *Homo sapiens* but also his autoimmune diseasome.

## Introduction

Molecular mimicry and the consequent potential cross-reactivity following infections have been repeatedly described in humans. 1 2 3 4 5 6 7 8 9 10 11 12 Such cross-reactivity is not evident in experimental infections of primates. 13 Actually, following preclinical studies performed in primates 14 15 16 17 18 as recommended by the Food and Drug Administration, 19 the reports declare that primate active immunization by pathogen vaccine administration is well tolerated and exempt of relevant events. Hence, the questions: why the potential cross-reactivity and the consequent potential autoimmune sequelae do not occur in primates following experimental infections or during preclinical tests? What are the genetic/phenetic determinants behind the different responses of humans and primates?

It was reasoned that, if it is true that molecular mimicry between infectious agents and human proteins contributes to or causes cross-reactions and postinfection autoimmune pathologies, then the human proteome should be characterized by different levels/patterns of molecular mimicry versus pathogens when compared with the proteomes of nonhuman primates. According to this rationale, this study comparatively analyzed primate and human proteomes for peptide sharing with pathogens by using heptapeptides as immunobiological units. 20 21 22 Analyses were also expanded to other mammalian species that are used in research laboratories (i.e., mice, rats, rabbits) and domestic animals such as dogs and cats. Results suggest that mice represent more suitable animal models for exploring potential autoimmune cross-reactions following pathogen administration and highlight new evolutionary scenarios in the origins of *Homo sapiens* .

## Materials and Methods

Peptide sharing analyses have been described elsewhere. 9 10 11 12 Briefly, pathogen proteomes (or proteins) were dissected into heptapeptides offset by one residue, and then each pathogen heptapeptide was analyzed for occurrences within proteomes from the following organisms (with National Center for Biotechnology Information [NCBI] TaxId in parentheses): *Homo sapiens* (9606); gorilla, *Gorilla gorilla gorilla* (9595); chimpanzee, *Pan troglodytes* (9598); and rhesus macaque, *Macaca mulatta* (9544). In addition, proteomes from the following mammalian organisms were analyzed as controls: cow, *Bos taurus* (9913); dog, *Canis lupus familiaris* (9615); cat, *Felis catus* (9685); rabbit, *Oryctolagus cuniculus* (9986); mouse, *Mus musculus* (10090); rat, *Rattus norvegicus* (10116); pig, *Sus scrofa* (9823); and bat, *Pteropus alecto* (9402).

The analyzed pathogen proteomes (or proteins) are (with NCBI TaxId in parentheses): poliovirus (12081); measles virus (70149); dengue virus (11059); severe acute respiratory syndrome-related coronavirus 2 (SARS-CoV-2) (2697049); hemagglutinin from influenza A virus, H1N1 (641809); major capsid protein L1 from human papillomavirus type 16 (333760); and protective antigen from *Bacillus anthracis* (1392).

Peptide matching analyses were conducted by using the PIR Peptide Matching program. 23

## Results

**Fig. 1:**
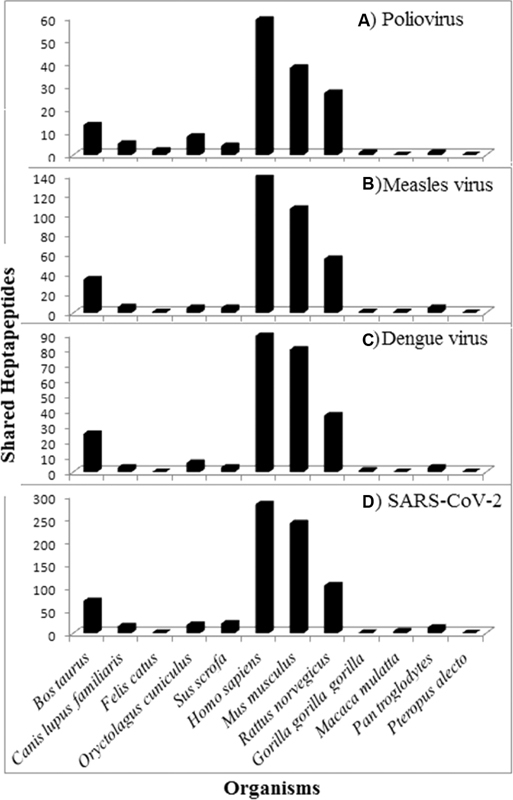
Heptapeptide sharing between mammalian proteomes and proteomes from: ( **A** ) poliovirus, ( **B** ) measles virus, ( **C** ) dengue virus, and ( **D** ) severe acute respiratory syndrome-related coronavirus 2 (SARS-CoV-2).

**Fig. 2:**
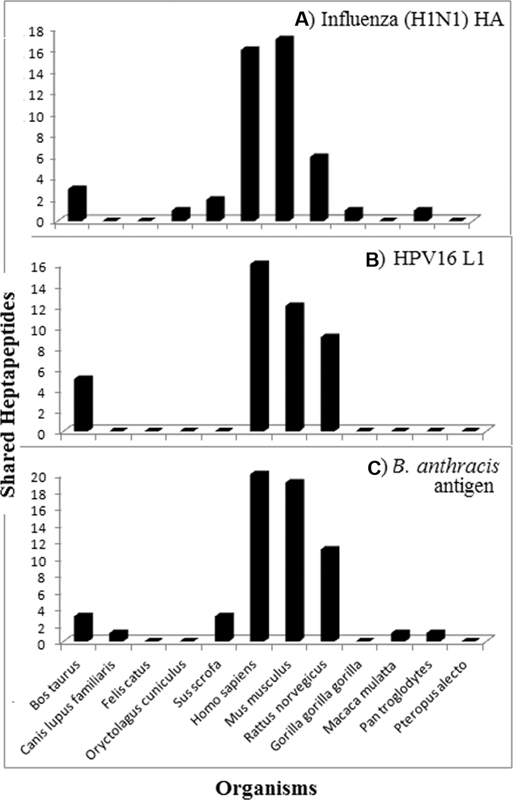
Heptapeptide sharing between mammalian proteomes and ( **A** ) hemagglutinin from influenza A virus, H1N1, ( **B** ) L1 from human papillomavirus type 16, and ( **C** ) protective antigen from *Bacillus anthracis* .

The heptapeptide sharing between the pathogen proteomes/proteins and the 12 mammalian proteomes is analytically detailed in Supplementary Tables S1–S7 (online only), and is graphically illustrated in Figs. 1 and 2 .

Specifically, Fig. 1 shows that:

- A massive heptapeptide sharing exists between the human proteome and poliovirus, measles virus, dengue virus, and SARS-CoV-2 proteomes.
- The viral heptapeptide sharing is likewise high with the murine proteome and, at a relatively lesser extent, with the rat proteome.
- In contrast, cat, dog, cow, pig, rabbit, and the three primates analyzed here have no or a low number of heptapeptides (see, for instance, cow) in common with the analyzed pathogens.
- Quantitatively, the highest number of peptide commonalities (namely, 281, see Supplementary Table S1 , online only) occurs between SARS-CoV-2 and *Homo sapiens* proteomes, while the bat *Pteropus alecto* was found to have no heptapeptide sequences in common with any of the analyzed pathogen proteomes/proteins.

Results similar to those reported in Fig. 1 are obtained when pathogen protein antigens (namely, influenza A HINI hemagglutinin, HPV16 L1, and *B. anthracis* protective antigen) are analyzed for peptide sharing with the 12 mammalian organisms ( Fig. 2 ). It can be seen that the shared heptapeptides occur in human, mouse, and rat proteomes, while primates and the other mammalian species remain almost completely excluded from the sharing or share a low number of heptapeptides.

## Discussion

Translational research requires animal models that allow researchers and clinicians to predict human responses. Nonhuman primates have long been fundamental research models for testing new therapies in clinical trials. Actually, this study documents that, when analyzing molecular mimicry, a highest number of pathogen heptapeptides occur in the human proteome but not in primate proteomes. As a logical consequence, this implies that the potential cross-reactive autoimmune effects of the pathogen versus human peptide overlapping cannot be evaluated in primates. In this regard, Figs. 1 and 2 indicate that only mice and rats and, at a minor extent, cows should be utilized in preclinical tests.

Our data are widely supported by clinical findings monitored during experimental infections. In summary:

- as reviewed by Wachtman and Mansfield, 13 natural poliovirus infection has not been diagnosed in rhesus macaques. Rhesus macaques are experimentally susceptible to poliovirus infection and develop encephalomyelitis if inoculated with poliovirus parenterally (usually intracranially). However, in many cases, no clinical signs are evident;
- likewise, measles disease is usually mild or asymptomatic in macaques, unless animals are stressed or immunosuppressed 23 ;
- experimental dengue infection of macaques and other monkeys produces viremia and antibody response but has been associated with only minimal clinical signs 24 ; and
- experimental SARS-CoV infections in primates do not reach the level of severity observed in human patients succumbing to respiratory failure. Indeed, SARS-CoV administered intranasally and intratracheally to rhesus, cynomolgus, and African green monkeys replicated in the respiratory tract but did not induce illness. 25

Then, the data illustrated in Figs. 1 and 2 appear of indisputable medical importance since scientifically substantiate what Hogan 26 clinically remarked, that is, the fact that the rhesus macaque model is of limited utility in preclinical tests, while only mice might represent a correct animal model for testing and evaluating immunotherapies to be used in humans. 27 28

Moreover, besides the medical relevance in the current experimental clinical context, the present study opens a new research perspective in the comparative study of humans and apes. Indeed, although the nucleotide difference between, for example, humans and chimpanzees is surprisingly small and amounts to a nucleotide difference of only 1 to 2%, on the other hand 80% of the proteins are different between the two species. 29 Understanding how the small nucleotide difference led to such a massive phenetic difference might be a key for understanding not only the fascinating evolutionary history of the origins of humans and apes, but also for investigating the basis of the autoimmune diseases that afflict humans, but not primates.

## Conclusion

Viruses appear to have played crucial roles in the viral eukaryogenesis process 30 31 as well as in the split between humans and apes million years ago. Indeed, the striking difference between primates and humans in the peptide sharing with pathogens not only indicates that primates are unreliable animal models for preclinical tests, but also highlights different phenotypic profiles that evolved along different evolutionary pathways. Such evolutionary pathways warrant further studies to understand primates' resistance to infections that are instead lethal in humans. Then, it seems that the role of the viruses in the cellular evolution of the human being has to be analyzed in a wider evolutionary and temporal context, where specific pathogen sequences were selectively incorporated into (or deleted from) the human and ape genomes. A comparative and multidisciplinary research approach appears to be mandatory to understand and fight the new and (re)emerging pathogens that violently threaten the human being, especially in the light of the massive peptide overlap between viral and human proteomes. 32 33
