# Supplementary material for: Medical, Genomic, and Evolutionary Aspects of the Peptide Sharing between Pathogens, Primates, and Humans
Source: Glob Med Genet. 2020 Aug 31;7(2):64–7. doi: 10.1055/s-0040-1716334 (PMC7490124; doi:10.1055/s-0040-1716334)
Supplement: Supplementary file 1 — Supplementary Material [file 10-1055-s-0040-1716334-s2000011.pdf]

Supplementary Appendix: Description of the Heptapeptide Sharing between Pathogens and Mammals

Supplementary Table S1 Poliovirus type 1 (strain Mahoney) (NCBI:taxid 12081)

Organism	N°	Shared heptapeptides
Bos taurus	13	EAIFSKY, EVEILDA, GFAAALK, LEALDLS, VEILDAK, ATLALLG, CPRPPRA, IITAGGE, PGAPVPE, QISDKIT, TKLRQLE, VTKLRQL, VLQLTLG
Canis lupus familiaris	5	LEALDLS, LLLKTYK, ATLALLG, CPRPPRA, QMLESMI
Felis catus	2	LEALDLS, QMLESMI
Gorilla gorilla gorilla	1	GDSLYGA
Homo sapiens	59	AAVAGVV, AGSAPLS, APLSVFT, CTGKVIG, EAIFSKY, EALDLST, EDAMYGT, EVDASLL, EVEILDA, GFAAALK, LEALDLS, LLLKTYK, LLLPEYS, LQAVDSQ, RALLLPE, SAPLSVF, VEILDAK, AAKGLEW, AALGDSL, ATLALLG, CPRPPRA, DGTLTPL, EKLLKNL, GAPVPEK, GDSLYGA, GTGKSVA, GTLTPLS, GVVIMDD, IITAGGE, KGLEWVS, KLLKNLI, LASTNSS, LATLALL, LGCDASP, PGTGKSV, PPGAPVP, QISDKIT, RPPRAVA, RSRSESS, RVYLKPK, SLPPDPS, SRISPPT, TKLRQLE, VNDHNPT, ATGKLLV, IVVPLST, KNTMEMY, LGNGTLL, LLGNAFV, LLGNGTL, LSLSPAS, PNTEASG, PREMDIL, PVDYLLG, QKALAQG, QMLESMI, TGKLLVS, VVPLSTP, YLRDSEA
Mus musculus	38	AGSAPLS, DASLSPA, EAIFSKY, EDAMYGT, EEAIFSK, EVDASLL, GFAAALK, GKSRLIE, IGRALLL, IYLFAGS, KEMQKLL, KSRLIEA, LEALDLS, LLLKTYK, LLLPEYS, LQAVTTF, MSLDINT, RALLLPE, AALGDSL, ALARRFA, ATLALLG, CPRPPRA, DGTLTPL, EILFNNV, FSDIRDL, GDSLYGA, GTLTPLS, IITAGGE, KDGTLTP, LATLALL, LYGAASL, PPGAPVP, QLEMLEN, RPPRAVA, SCPSQEH, SLEEKGI, TKLRQLE, VPPGAPV
Oryctolagus cuniculus	8	AAVAGVV, EAIFSKY, EVDASLL, AALGDSL, CPRPPRA, TKLRQLE, LPLAPLN, QMLESMI
Pan troglodytes	1	GDSLYGA
Rattus norvegicus	27	EAIFSKY, EDAMYGT, EVDASLL, FHYVFEG, GFAAALK, GLPNKKP, IGRALLL, LEALDLS, LLLKTYK, ALARRFA, ATLALLG, CPRPPRA, EILFNNV, FTSNYVL, IITAGGE, KDTVQLR, LATLALL, LYGAASL, QISDKIT, RSRSESS, APPGADP, ATGKLLV, LLGNGTL, PVDYLLG, QMLESMI, VVPLSTP, YLRDSEA
Sus scrofa	4	EALKMVL, GFAAALK, LEALDLS, CPRPPRA

Supplementary Table S2 Measles virus (strain Edmonston-Zagreb vaccine) (NCBI:taxid 70149)

Organism	N°	Shared heptapeptides
Bos Taurus	34	ACQVIAE, ALLPAPI, KDKALAA, LARALRS, LASLMPE, LLGLGVL, NVSLEEL, QLARALR, SLEELRV, SQSLKSI, AVLLTLQ, EIAEYRR, EPGEDGL, KDTIEKL, LLNEELE, LLSSFLS, VGLVEHR, VLLTLQT, DVE-TAEG, GPGAPAG, PPPPDPG, SLSLLDL, SSNLVIL, VLTPLFK, DVETAEG, GLSRPSP, IKKGHRR, IPLDTPQ, KLESLLL, LDTPQRF, LESLLLL, PPPPDPG, SEVYSAD, VFALGGI
Canis lupus familiaris	6	EAVTAAE, LGSELRE, LREDIKE, VSLEELR, KDTIEKL, ALIQEWT
Felis catus	1	GPASRSV
Gorilla gorilla gorilla	1	GLGDRKD
Homo sapiens	140	ACQVIAE, ALAALQR, ALLPAPI, CHRRRHT, CSVQLAR, DKESTRK, EAVTAAE, ELLISRD, ELRAELC, ESMRGHL, GIYYDGL, GSRRLVD, GTGSRRL, HPRLEAV, IRASMRK, LAVSGVP, LLGLGVL, LLVSQSL, LNDSSFD, LREDIKE, NAQASGE, PDVLESM, PSRSLRS, RLARGRP, RLEAVTA, SELREDI, SLEELRV, SLFAQRL, SPVHTST, SQSLKSI, SRLGLGS, SRRLVDV, SVELLIS, TCHRRRH, TGRKRNV, TKGLIRA, TSLVRVA, TVELRGA, VINLGVY, AEVNVSQ, AVLLTLQ, CSLTYLR, DLIGQKL, EIAEYRR, EYRRLLR, GEDGLFL, GIKARIT, GPSLRDP, HCGIIEP, KDTIEKL, KLEELAA, KLVGYSA, LCKELIH, LEKLGYS, LIGQKLG, LLNEELE, LLSSFLS, LNEELEE, LRASLET, LSPAGSS, LTLQTPT, NIFVKNL, PVLVSSR, RELISRI, RVDPGFI, SSRQREL, SSVRTSP, VGLVEHR, VLLTLQT, AGSSGLS, AKELLES, ASLLTGG, DNESENS, DVETAEG, EELMNAL, EISDNPG, ELSQLSM, FLSLIGL, GFGPLIT, GLLAIAG, GPGAPAG, HELLRLQ, KELLESS, KRSELSQ, LEDAKEL, LGELKLA, LLAIAGI, NLDVTNS, PGLGAPV, RLHRAAI, RSMKGLS, SDVDIGE, SELSQLS, SGFGPLI, SGPGAPA, SLSLLDL, SNMSLSL, SSNLVIL, VLFVMFL, VLLAVLF, VLTPLFK, YPDAVYL, ADVEINP, AGSSGLS, AVKRLRE, AVLQPSV, DLERAMT, DNESENS, DNGYYTV, DVEINPD, DVETAEG, EISDNPG, FLLGVVE, GLGDRKD, GPASRSV, GRALAEV, HELLRLQ, HIRSTGK, IPGLGKD, ISISTLE, KLESLLL, LAKIHED, LESLLLL, LKEATEL, LLKEATE, LLKEFQL, LSRPSPS, QKEGRST,

(*Continued*)

Supplementary Table S2 (*Continued*)

Organism	N°	Shared heptapeptides
		RQAVRVS, SDVDIGE, SGLSRPS, SKLESLL, SKMLTLS, SLTGDLL, SRPSPSA, SSRLEED, SVIRSII, VFALGGI, VKRLRES, VRIQAVL
Macaca mulatta	1	GLGDRKD
Mus musculus	106	ACQVIAE, ALAALQR, ALLPAPI, DPHCPPD, EAVTAAE, EGYCQKL, ELRAELC, ESMRGHL, GIYYDGL, GSRRLVD, GSSNTVL, GTGSRRL, IPSSRKL, KRLETSV, LLGLGVL, LLVSQSL, NVSLEEL, PDVLESM, PSRSLRS, SDLTMYL, SLEELRV, SLKEKEI, SQSLKSI, SRRLVDV, SRSLRSA, SSVELLI, SVELLIS, SVLYVSD, TGRKRNV, TGSSNTV, VELLISR, VINLGVY, YLTFELV, AVLLTLQ, CSLTYLR, DLIGQKL, EELAAIL, EIAEYRR, EYRRLLR, HCGIIEP, HHDVASG, KDTIEKL, LAGAALG, LEKLGYS, LEPGEDG, LRASLET, PGEDGLF, PVLVSSR, SILVIKL, SSACYKA, SSRQREL, TAGIALH, VGLVEHR, VLLTLQT, AGSSGLS, ASFGTEI, DGDVKLS, DVETAEG, EELMNAL, EISDNPG, FLSLIGL, GFGPLIT, GKGVSFQ, GPGAPAG, GSSGLSK, HELLRLQ, HGSGMDL, LEDAKEL, LGELKLA, PPPPDPG, REEKAGS, RLHRAAI, SGFGPLI, SGPGAPA, SLSLLDL, SSNLVIL, STDDPVI, VLLAVLF, VLTPLFK, VRSLMSP, YGVLSVD, YVLLAVL, AGSSGLS, AVLQPSV, DNGYYTV, DVETAEG, EISDNPG, EVYSADY, FLLGVVE, GPASRSV, GRALAEV, GSSGLSK, HELLRLQ, ISKLESL, KSSRLEE, LKEATEL, LLKEATE, LLKEFQL, LPSLWGS, PPPPDPG, QLPEATF, REEKAGS, RTSSRGQ, SKLESLL, SKMLTLS, STAKPEK
Oryctolagus cuniculus	5	LRGAFLN, EIAEYRR, GFGPLIT, SGFGPLI, GPASRSV
Pan troglodytes	5	KEEDEGL, RELISRI, SNMSLSL, GLGDRKD, SLTGDLL
Rattus norvegicus	55	ALLPAPI, EAVTAAE, ESMRGHL, GIYYDGL, GSSNTVL, GTGSRRL, IPSSRKL, LLGLGVL, PDVLESM, QGMLLGL, SLEELRV, SQSLKSI, STRNVRA, TGRKRNV, TPISTST, TVELRGA, VINLGVY, VYSKGIY, YLTFELV, EIAEYRR, GPSLRDP, KDTIEKL, LAGAALG, PVLVSSR, VLLTLQT, VVLAGAA, YLDLLLN, YYTEILS, DGDVKLS, DVETAEG, EELMNAL, EISDNPG, GFGPLIT, GKGVSFQ, GLSSTSI, GPGAPAG, KGLSSTS, PPPPDPG, SGFGPLI, SLSLLDL, VLTPLFK, YGVLSVD, DNGYYTV, DVETAEG, EISDNPG, GPASRSV, KLESLLL, KSSRLEE, LLKEFQL, LPSLWGS, PPPPDPG, QLKPIGK, QLPEATF, RQAVRVS, SKMLTLS
Sus scrofa	5	EVPDVLE, LLGLGVL, EIAEYRR, GDLLGIL, SEVYSAD

Supplementary Table S3 Dengue virus type 1 (strain Nauru/West Pac/1974) (NCBI:taxid 11059)

Organism	N°	Shared heptapeptides
Bos taurus	25	AGLAFSL, AKRGTAQ, DLGCGRG, GAGWSLR, GEKWKRQ, QAAILMG, RDLRLAA, CVTKLEG, KGLLSGQ, KYSVIVT, LTGATEI, RGKSLLF, AMILSIV, EVDSFSL, GEVDSFS, ILLSSLL, KIKDEER, LLSSLLK, PVLLGSL, TTNIWLK, EGEVGAI, GKEIVDL, IIMDEAH, LSGRGLG, LVLAPTR
Canis lupus familiaris	3	QAAILMG, ASTSQET, LLLTVGL
Gorilla gorilla gorilla	1	EHGTTAT
Homo sapiens	89	AGAGLAF, AGLAFSL, AKRGTAQ, DLGCGRG, ERELHKQ, ETLEQMQ, GAGWSLR, LFMILTA, LLFMILT, NTTANIS, PSGSASS, QAAILMG, RDLRLAA, SLIGLTA, TLATGPL, TNIQVAI, VAVEPEV, VDLHPAS, VMDVISR, YADDTAG, AGEKALK, ALTLDCS, EHGTTAT, FLAIPPT, GAGEKAL, GKIVQYE, GKMFEAT, GLSGATW, GSLITCA, KGLLSGQ, KMFEATA, LGSQEGA, LLMLLPT, NLKYSVI, PAVLRKL, RGKSLLF, VSQLAKR, VVLGSQE, YGTCSQT, YIVVGAG, ADLSLEK, DVSGILA, ELGDGLA, EVEDYGF, FAVGLLF, GDVSGIL, GIVSILL, GLLFRRL, GSGIFVT, HLGKLEL, ILLSSLL, KIWGRKS, KLELDFD, LGKLELD, LIHQIFG, LLKATLL, LLLTMGQ, LLSLTFV, LPNSLEE, LSLVASV, LTSREVL, NGVLESE, PSLRTTT, PVLLGSL, SSADLSL, VLLLTVG, DFPGKTV, EGEVGAI, EREKSAA, GKEIVDL, IIMDEAH, KPGTSGS, KRVVQLS, LKPVILK, LLSPVRV, LREFKEF, LVLAPTR, PGTSGSP, PPGSVEA, PSPPEVE, PVTVASA, RLRGEAR, SPPEVER, TEAKMLL, TLMLLAL, TPSPPEV, VGLYGNG, VRTLVLA, VTVASAA
Mus musculus	80	AGAGLAF, AKRGTAQ, CLGKSYA, ESDPEGA, FEKQLGQ, FSPSELE, GDDCVVK, GETLGEK, LFMILTA, LLATSIF, LLFMILT, MEAQLIR, NMEAQLI, NTTANIS, QAAILMG, RDLRLAA, VAVEPEV, VDLHPAS, YADDTAG, AGEKALK, ALTLDCS, AMDLGEL, EVVVLGS, GKIVQYE, GKMFEAT, GLSGATW, KGLLSGQ, KMFEATA, LGSQEGA, LLMLLPT, LTGATEI, LVMAFIA, QEVVVLG, TQNGRLI, AEHSGAS, AGPWHLG, CLSTTSQ, EVEDYGF, FAVGLLF, GLLFRRL, GLSLVAS, GPSLRTT, GSGIFVT, GSIGGVF, HLGKLEL, ILLSSLL, KLELDFD, LGCKPLT, LGKLELD, LLKATLL, LLLTVGL, LSLVASV, LSSLLKN, LTSREVL, PSLRTTT, SILLSSL, SNGVLES, SSLLKND, TVGLSLV, DPRRCLK, EGEVGAI, ERAVLDD, EREKSAA, ERKKLRP, GKEIVDL, HPGSGKT, IVREAIR, KEGERKK, LLSPVRV, LREFKEF, LVLAPTR, PPGSVEA, QEGPLPE, RLRGEAR, TPSPPEV, VGLYGNG, VREAIRR, VRTLVLA, VTVASAA, YVVTTDI
Oryctolagus cuniculus	6	AGAGLAF, KRQLNQL, GSGIFVT, GMGEAAA, LVLAPTR, QRGLLGR
Pan troglodytes	3	EHGTTAT, YSVIVTV, LVLAPTR

Supplementary Table S3 (*Continued*)

Organism	N°	Shared heptapeptides
Rattus norvegicus	37	AGAGLAF, AKRGTAQ, CLGKSYA, LAANAIC, LFMILTA, LLFMILT, NTTANIS, QAAILMG, RDLRLAA, SLIGLTA, TTLWEGS, VAVEPEV, YAVATTI, AGEKALK, AKISNTT, GKMFEAT, KMFEATA, LGSQEGA, AEHSGAS, CLSTTSQ, GLSLVAS, GSGIFVT, GSIGGVF, HLGKLEL, LGKLELD, LSIVSLF, LTSREVL, PSLRTTT, QLWATLL, GKEIVDL, IVREAIR, LVLAPTR, MRLLSPV, TPSPPEV, VREAIRR, VTVASAA, YVVTTDI
Sus scrofa	3	QAAILMG, QNKVVRV, RDLRLAA

Supplementary Table S4 Severe acute respiratory syndrome coronavirus 2 (SARS-CoV-2) (NCBI:taxid 2697049)

Organism	N°	Shared heptapeptides
Bos taurus	70	DFTEERL, AMQRKLE, ASAVVLL, AVVLLIL, EKMVSLL, LFLLPSL, LGVGGKP, NYEPLTQ, QARSEDK, SLLSVLL, YNYEPLT, ADVEWKF, ASKILGL, DPAQLPA, EYFNSVC, FEKGDYG, GPPGTGK, HISTIGV, NVAITRA, PPGTGKS, RYPANSI, VAITRAK, VDLFRNA, VLSNLNL, YPANSIV, EILQKEK, ESGLKTI, GAKLKAL, KDLLARA, KGKAKKG, SEARQHL, SGLKTIL, EKLKTLV, IALKGGK, KGGKIVN, LIWNVKD, TLATHGL, TSNSFDV, VDNSSLT, FKNLREF, FVSDADS, KVVKVTI, LLDDFVE, PQTLEIL, QTLEILD, TRAGCLI, LDKYFKN, LLLVAAG, LVKQLSS, RVAGDSG, SHLLLVA, SSTASAL, TTTSVPL, AALALLL, AEGSRGG, ALALLLL, DAALALL, GKMKDLS, LALLLLD, PSASAFF, SRSSSRS, EEEQEED, EVKPFIT, IPKEEVK, KHGGGVA, LKHGGGV, LLAPLLS, NLKTLLS, PEEEQEE, VVADAVI
Canis lupus familiaris	15	APGTAVL, GPPGTGK, KGKAKKG, LKHGGGV, IALKGGK, VDNSSLT, DTSLSGF, EKMVSLL, VAGGIVA, ALITLAT, LALITLA, LLLVAAG, SVLLFLA, TVEELKK, VFLLVTL
Felis catus	1	DTSLSGF
Gorilla gorilla gorilla	1	LKTLLSL, ALITLAT, LALITLA
Homo sapiens	281	DFTEERL, DTLKEIL, GGTSSGD, GNLLLDK, GSGVPVV, GTSSGDA, HAASGNL, HQKLLKS, ILTLTRA, KLVLSVN, LGERVRQ, LLSDLQD, PGSGVPV, PLVRKIF, QDALFAY, RARTVAG, RLSFKEL, RQLLFVV, SRQRLTK, SSGDATT, TVLQAVG, VDAAKAY, VRQALLK, YGCSCDQ, AGKASCT, EEIAIIL, EEVVLKT, EIKDTEK, ESGLKTI, EVEKGVL, EVLTEEV, FIKRSDA, GAKLKAL, GETLPTE, KDLLARA, KFLALCA, KGKAKKG, LARAGKA, LASFSAS, LEILQKE, LVAELEG, PQLEQPY, RVEKKKL, SCGNFKV, SEARQHL, SGLKTIL, SVEEVLS, TEEVVLK, TENLTKE, VEKGVLP, APGTAVL, CVCSVID, DEVRQIA, DKVFRSS, FKNLREF, FLPFFSN, FVSDADS, GRLIIRE, IGAGICA, IQQKLAL, KLNDLCF, KTKNVTK, KVVKVTI, LDDFVEI, LHLLIGL, LSLLSKG, PGTAVLR, QRMLLEK, RARSVAS, RRARSVA, SLLIVNN, SPRRARS, SSSGWTA, TLIGEAV, TRFQTLL, VSGTNGT, VTLIGEA, VYSTGSN, AALALLL, AEGSRGG, ALALLLL, DAALALL, DKKKKAD, FLVFLGI, FTLKRKT, GKMKDLS, KADETQA, KDKKKKA, KKDKKKK, KLGSLVV, LGIITTV, LLLLDRL, LLPAADL, LVFLGII, NSSPDDQ, RGQGVPI, RPQGLPN, RQEEVQE, RSSSRSR, SKQLQQS, SMSSADS, SRGGSQA, SRSRNSS, SRSSSRS, SSRSSSR, VAAIVFI, VLQLPQG, ADVEWKF, AENVTGL, APTLVPQ, DIPGIPK, EYFNSVC, FNVAITR, GKPRPPL, GPPGTGK, HISTIGV, IATVREV, IPGIPKD, IVDTVSA, LFAAETL, LQGPPGT, NVAITRA, PEVKILN, PPGTGKS, QGPPGTG, RFNVAIT, RPQIGVV, RYPANSI, SKILGLP, TREAVGT, TRLQSLE, TVDSSQG, VAITRAK, VCNAPGC, VDLFRNA, VNALPET, YPANSIV, AIFYLIT, AKKNNLP, DSDVETK, EKLKTLV, ELSRVLG, FDSEYCR, GDFLHFL, GLAAVNS, IALKGGK, IIWFLLL, IKVTLVF, KGGKIVN, KKGAKLL, KKNNLPF, LACEDLK, LAKNVSL, LSLQFKR, NSLKITE, PGTILRT, QPILLLD, RFFYVLG, RRSFYVY, RSFYVYA, RYVLMDG, SAKSASV, SIHLYFD, TLATHGL, TPSFKKG, TSNSFDV, VDNSSLT, VTVKNGS, ALITLAT, DEDDSEP, EDDSEPV, EELKKLL, EIDRLNE, ELKKLLE, GLTVLPP, HLLLVAA, KCRSKNP, KVEAEVQ, LAFVVFL, LAILTAL, LALITLA, LDKYFKN, LIRAAEI, LKKLLEQ, LLESELV, LLLVAAG, LLVAAGL, LPPLLTD, LVKQLSS, NASVVNI, NSVLLFL, SSTASAL, SVLLFLA, SVTSSIV, TALRLCA, TGRLQSL, TQLSTDT, VEELKKL, VFLLVTL, VGVALLA, VNSVLLF, VTLADAG, AGEAANF, ATALLTL, DEDEEEG, DIQLLKS, EDEEEGD, EDIQLLK, EEEQEED, EGKTFYV, ELTPVVQ, EVKPFIT, GPLKVGG, IPKEEVK, IQLLKSA, KEEVKPF, KLVSSFL, KTTVASL, LGIEFLK, LKGVEAV, LKSAYEN, LKTLLSL, LLAPLLS, LLSAGIF, LSGHNLA, NDLNETL, NGNLHPD, NLKTLLS, PDEDEEE, PEEEQEE, PEEHFIE, SAALQPE, SELLTPL, SKTTVAS, TFDNLKT, TLQQIEL, TLVSDID, VSELLTP, VSVSSPD, YENFNQH, AACCHLA, AMQRKLE, ASAVVLL, AVVLLIL, DSEVVLK, DTSLSGF, DVVYCPR, EKMVSLL, EVVLKKL, FTPLIQP, GTTFTYA, GVVTTVM, IASEFSS, KELLQNG, LEGNFYG, LFLLPSL, LKELLQN, LKKLKKS, LLPLTQY, LTSLLVL, NGRTILG, NSGSDVL, NYEPLTQ, PSLATVA, QARSEDK, QGLLPPK, SASIVAG, SGVVTTV, SGVYQCA, SLKELLQ, SLLSVLL, SLLVLVQ, SVVLLSV, TDTTITV, TILTSLL, TSLLVLV, TSLSGFK, VAGGIVA, VLKLKVD, VSLLSVL, YNYEPLT
Macaca mulatta	4	APTLVPQ, ELTPVVQ, TLEILDI, YDTNVLE
Mus musculus	240	AALALLL, ALALLLL, ASSRSSS, DAALALL, FTLKRKT, ITTVAAF, KKDKKKK, KLGSLVV, LALLLLD, LVFLGII, RPQGLPN, RSSSRSR, SRSRNSS, SRSSSRS, SSRSSSR, TGAIKLD, VGARKSA, VLQLPQG, DFTEERL, GSGVPVV, HQKLLKS, ILTLTRA, LLSDLQD, NICQAVT, NLLLDKR, PGSGVPV, QDALFAY, QEKDEDD, QKLLKSI, RARTVAG, RLSFKEL, RQLLFVV, TVLQAVG, VDAAKAY, YGCSCDQ, YKDYLAS, AELEGIQ,

(*Continued*)

Supplementary Table S4 (*Continued*)

Organism	N°	Shared heptapeptides
		DNLLEIL, EIKDTEK, ESGLKTI, EVEKGVL, FIKRSDA, GETLPTE, GVVGEGS, KDLLARA, KGKAKKG, KPVLDWL, LEILQKE, LPTEVLT, LVAELEG, PACHNSE, PLVGTPV, PQLEQPY, SEARQHL, SGLKTIL, TEEVVLK, TENLTKE, TLPTEVL, VEKGVLP, AALLADK, ADVEWKF, ANEYRLY, CTERLKL, DIPGIPK, EVKILNN, FNVAITR, GPPGTGK, HISTIGV, IATVREV, IVDTVSA, LFAAETL, NVAITRA, PEVKILN, PPGTGKS, QGPPGTG, RFNVAIT, RYPANSI, SKILGLP, TRLQSLE, VDLFRNA, VKILNNL, VNLKQLP, YPANSIV, AKNLNES, ALITLAT, EAEVQID, EIDRLNE, ELKKLLE, FVVFLLV, GLTVLPP, HSSSSDN, INLIIKN, LAILTAL, LALITLA, LFLAFVV, LIRAAEI, LKGCCSC, LKKLLEQ, LLLVAAG, LLVAAGL, LVKQLSS, NHTSPDV, NSVLLFL, RPLLESE, SSTASAL, SSVLNDI, SVLLFLA, TGRLQSL, VEELKKL, VGVALLA, VLLFLAF, VNSVLLF, CTEVPVA, DEVRQIA, DSFVIRG, EAVKTQF, ENGTITD, FIERYKL, FKNLREF, FLVLLPL, FVSDADS, GRLIIRE, GSVAIKI, IDLLLDD, IQQKLAL, IVYGDFS, KVVKVTI, LIVNNAT, LLPLVSS, NCTEVPV, NPIQLSS, NRKRISN, PGTAVLR, QPGVAMP, QTLEILD, RKSNLKP, SFVIRGD, SSSGWTA, TLIGEAV, TLLALHR, TRFQTLL, VTKENDS, VTLIGEA, AKKNNLP, ASGKPVP, AYVNTFS, CLSGLDS, ESSAKSA, FDAYVNT, GGVTRDI, IALKGGK, IKVTLVF, KGGKIVN, KKNNLPF, LACEDLK, LAKNVSL, LKTLVAT, NSLKITE, PGTILRT, QPILLLD, RFFYVLG, RRSFYVY, RSFYVYA, RVVTTFD, SAKSASV, SLSEQLR, STFISAA, TKGSLPI, TLATHGL, TSNSFDV, VDNSSLT, VLEGSVA, VTRDIAS, VTVKNGS, VVTTFDS, AMQRKLE, AVVLLIL, DVVYCPR, EKMVSLL, EVVLKKL, FNTLLFL, GPLSAQT, GTTFTYA, IASEFSS, IVTALRA, LFLLPSL, LGVGGKP, LKELLQN, LLPLTQY, LLPPKNS, LTSLLVL, NSGSDVL, QAR-SEDK, QGLLPPK, QLRVESS, SASIVAG, SGVVTTV, SLLSVLL, SVVLLSV, TAQEAYE, TDTTITV, VAGGIVA, VESSSKL, VLKLKVD, VSFLAHI, VSLLSVL, YNYEPLT, AGEAANF, DEDEEEG, DIQLLKS, EDEEEGD, EEEQEED, EGKTFYV, HGGGVAG, IKIQEGV, IPKEEVK, IQLLKSA, KTTVASL, LEFGATS, LGIEFLK, LKGVEAV, LKSAYEN, LKTLLSL, LLAPLLS, LLSLREV, LSGHNLA, MLAKALR, NGNLHPD, NLKTLLS, PEEEQEE, PEEHFIE, PLLSAGI, SKTTVAS, SYSGQST, TESKPSV, TFDNLKT, TKKAGGT, VSELLTP, VSVSSPD, VVVNAAN, YENFNQH, YTVEEAK
Oryctolagus cuniculus	18	AALTNNV, IVYGDFS,EDIQLLK, SAALQPE, FIKRSDA, KGKAKKG,KDFMSLS, VDNSSLT, EIDRLNE, LIFLWLL, LLLVAAG, EKMVSLL, SGAMDTT, VAGGIVA, EVKILNN, GPPGTGK, PEVKILN, VKILNNL
Pan troglodytes	12	APTLVPQ, FIKRSDA, LEGNFYG, TSNSFDV, YGCSCDQ, EDEEEGD, LKTLLSL, RARSVAS, RRARSVA, ALITLAT, EELKKLL, LALITLA
Rattus norvegicus	104	FKNLREF, FVSDADS, IVYGDFS, KVVKVTI, LLPLVSS, PGTAVLR, SSVLHST, TLIGEAV, VSGTNGT, VTLIGEA, AALALLL, ALALLLL, DAALALL, KKDKKKK, KLGSLVV, LLLLDRL, PPTEPKK, RSSSRSR, SRSSSRS, SSRSSSR, TGAIKLD, VLQLPQG, ALDISAS, AVVLLIL, EKMVSLL, GPLSAQT, LGSALLE, LGVGGKP, LLPLTQY, QARSEDK, QEAYEQA, SLATVAY, VAGGIVA, YNYEPLT, ALITLAT, EIDRLNE, LALITLA, LFLAFVV, LIRAAEI, LKGCCSC, LKKLLEQ, LLLVAAG, LSKSLTE, SSDNIAL, SSTASAL, VNSVLLF, ADVEWKF, ETLKATE, EYFNSVC, GPPGTGK, HISTIGV, IVDTVSA, NVAITRA, PPGTGKS, RYPANSI, SYATHSD, VAITRAK, VDLFRNA, YPANSIV, ATCEFCG, ETVKGLD, EVEKGVL, FIKRSDA, FSASTSA, GAKLKAL, KGKAKKG, KLKALNL, LEILQKE, PACHNSE, SEARQHL, TLPTEVL, VEKGVLP, DEDEEEG, DIQLLKS, EEEQEED, EVKPFIT, ILPSIIS, IPKEEVK, IQLLKSA, KTTVASL, LKSAYEN, LKTLLSL, LSGHNLA, NGNLHPD, NLKTLLS, PEEEQEE, PEEHFIE, YTVEEAK, LVKQGDD, QEKDEDD, RARTVAG, STDVVYR, VDAAKAY, YGCSCDQ, YQHEETI, FCLEASF, IALKGGK, IKVTLVF, KGGKIVN, RFFYVLG, RRSFYVY, RSFYVYA, TSNSFDV, VDNSSLT
Sus scrofa	21	DFTEERL, GPPGTGK, IQDSLSS, EKMVSLL, TTSYREA, IALKGGK, VDNSSLT, KGKAKKG, VETVKGL, AALALLL, SASAFFG, SRSSSRS, FVSDADS, TLIGEAV, VTLIGEA, EDEEEGD, EVKPFIT, KEEVKPF, KHGGGVA, LKHGGGV, NLKTLLS, PEEEQEE

Supplementary Table S5 Hemagglutinin from influenza A virus (H1N1) (NCBI:taxid 641809)

Organism	N°	Shared heptapeptides
Bos taurus	3	GAINTSL, VASSLVL, YDYPKYS
Gorilla gorilla gorilla	1	AKLNREE
Homo sapiens	16	AELLVLL, AKLNREE, EELREQL, ELLVLLE, ELREQLS, GAINTSL, GVAPLHL, GVKLEST, KVRDQEG, LREQLSS, LRLATGL, MKAILV, VKLESTR, VRDQEGR, YDYPKYS, YVKSTKL
Mus musculus	17	AADLKST, AELLVLL, AKLNREE, EELREQL, EKNVTVT, ELLVLLE, GAINTSL, GVKLEST, LIWLVKK, LREQLSS, PSTSADQ, QLSSVSS, TVLEKNV, TYNAELL, VASSLVL, YDYPKYS, YVKSTKL
Oryctolagus cuniculus	1	AKLNREE
Pan troglodytes	1	DLKSTQN
Rattus norvegicus	6	AELLVLL, EELREQL, GAINTSL, LREQLSS, VASSLVL, YDYPKYS
Sus scrofa	2	ASSLVLV, YDYPKYS

Supplementary Table S6 Major capsid protein L1 from human papillomavirus type 16 (NCBI:taxid 333760)

Organism	N°	Shared heptapeptides
Bos taurus	5	ATPTTSS, KSEVPLD, LQPPPGG, TSSTSTT, TTSSTST
Homo sapiens	16	AAISTSE, AGTSRLL, AVGENVP, EKFSADL, GSGSTAN, KATPTTS, KGSGSTA, KSEVPLD, LQFIFQL, LQPPPGG, PGGTLED, PTPSGSM, PVPVSKV, QLFVTVV, TLQANKS, VPKVSGL
Mus musculus	12	AGTSRLL, ATPTTSS, EKFSADL, GLQPPPG, GSGSTAN, KGSGSTA, KSEVPLD, LPPVPVS, LQPPPGG, PTTSSTS, QLFVTVV, QRAQGHN
Rattus norvegicus	9	ATPTTSS, FPTPSGS, GLQPPPG, GSGSTAN, KSEVPLD, LQPPPGG, SSTSTTA, STSTTAK, VAVNPGD

Supplementary Table S7 Protective antigen from *Bacillus anthracis* (NCBI:taxid 1392)

Organism	N°	Shared heptapeptides
Bos taurus	3	DNDGIPD, LPLYISN, PSENGDT
Canis lupus familiaris	1	RLLNESE
Homo sapiens	20	AKENQLS, DNDGIPD, ELENIPS, EVIQAEV, INKASNS, KLYWTDS, LELEKTK, LKEALKI, LPLYISN, LSIPSSE, NQLAELN, PLMALST, PLYISNP, QRENPTE, RFHYDRN, RLLNESE, SSELENI, TSSTTGD, TSTNGIK, VLPTTSL
Macaca mulatta	1	RLLNESE
Mus musculus	19	DNDGIPD, EVIQAEV, KLYWTDS, KRSTSAG, LEKTKQL, LKEALKI, LSIPSSE, PLMALST, PSDPLET, PTEKGLD, RLLNESE, SLAGERT, SNSNSST, SSELENI, SSTGNLE, TARIIFN, TLKEALK, TSTNGIK, VGADESV
Pan troglodytes	1	RLLNESE
Rattus norvegicus	11	EVIQAEV, KLYWTDS, KRSTSAG, LEKTKQL, LSIPSSE, MLNISSL, PLMALST, PSKNLAP, PTEKGLD, SKNTSTS, SSELENI
Sus scrofa	3	ISKNTST, NSSTEGL, RLLNESE
